# Endurance training increases the efficiency of rat skeletal muscle mitochondria

**DOI:** 10.1007/s00424-016-1867-9

**Published:** 2016-08-27

**Authors:** Jerzy A. Zoladz, Agnieszka Koziel, Andrzej Woyda-Ploszczyca, Jan Celichowski, Wieslawa Jarmuszkiewicz

**Affiliations:** 1 Department of Muscle Physiology, Chair of Physiology and Biochemistry, Faculty of Rehabilitation, University School of Physical Education, 31-571 Krakow, Poland; 2 Department of Bioenergetics, Adam Mickiewicz University, 61-614 Poznan, Poland; 3 Department of Neurobiology, University School of Physical Education, 61-871 Poznan, Poland

**Keywords:** Endurance training, Skeletal muscle mitochondria, Temperature, Oxidative phosphorylation, Uncoupling protein, Mitochondria functioning, Reactive oxygen species

## Abstract

Endurance training enhances mitochondrial oxidative capacity, but its effect on mitochondria functioning is poorly understood. In the present study, the influence of an 8-week endurance training on the bioenergetic functioning of rat skeletal muscle mitochondria under different assay temperatures (25, 35, and 42 °C) was investigated. The study was performed on 24 adult 4-month-old male Wistar rats, which were randomly assigned to either a treadmill training group (*n* = 12) or a sedentary control group (*n* = 12). In skeletal muscles, endurance training stimulated mitochondrial biogenesis and oxidative capacity. In isolated mitochondria, endurance training increased the phosphorylation rate and elevated levels of coenzyme Q. Moreover, a decrease in mitochondrial uncoupling, including uncoupling protein-mediated proton leak, was observed after training, which could explain the increased reactive oxygen species production (in nonphosphorylating mitochondria) and enhanced oxidative phosphorylation efficiency. At all studied temperatures, endurance training significantly augmented H_2_O_2_ production (and coenzyme Q reduction level) in nonphosphorylating mitochondria and decreased H_2_O_2_ production (and coenzyme Q reduction level) in phosphorylating mitochondria. Endurance training magnified the hyperthermia-induced increase in oxidative capacity and attenuated the hyperthermia-induced decline in oxidative phosphorylation efficiency and reactive oxygen species formation of nonphosphorylating mitochondria via proton leak enhancement. Thus, endurance training induces both quantitative and qualitative changes in muscle mitochondria that are important for cell signaling as well as for maintaining muscle energy homeostasis, especially at high temperatures.

**Electronic supplementary material:**

The online version of this article (doi:10.1007/s00424-016-1867-9) contains supplementary material, which is available to authorized users.

## Introduction

It was originally demonstrated by Holloszy [20] that endurance training enhances the synthesis of muscle mitochondrial proteins and increases their activities in rat skeletal muscles within a few weeks. A similar response to endurance training was confirmed in human skeletal muscles [21, 30]. Training-induced intensification of mitochondrial biogenesis is thought to be responsible for an enhancement of muscle metabolic status during exercise and an increased resistance to fatigue [13]. Mitochondria are also an important source of reactive oxygen species (ROS) formation [31]. Enhanced ROS level can be involved not only in cell damage [1, 8] but also in vital processes maintaining muscle homeostasis and muscle adaptation to exercise, including mitochondria biogenesis [22]. Surprisingly, little is known regarding the effect of endurance training on qualitative regulatory changes within mitochondria, such as mitochondrial membrane potential (mΔΨ), oxidative phosphorylation (OXPHOS), efficiency (ADP/O ratio), proton leak, or ROS production (for review see [4]).

It has been demonstrated that acute changes in assay temperature can influence the ADP/O ratio in isolated skeletal muscle mitochondria [6, 25, 38]. Our recent study extended the knowledge in this area by showing that, for nonphosphorylating rat skeletal muscle mitochondria, increasing the assay temperature from 25 to 42 °C leads to a decrease in membrane potential, hydrogen peroxide production, and coenzyme Q (quinone (Q)) reduction level [25]. Moreover, an increase in proton leak, including uncoupling protein-mediated proton leak, was observed with an increase in assay temperature, which could explain the reduced oxidative phosphorylation efficiency and ROS production. Although the efficiency of oxidative phosphorylation decreased, the oxidation and phosphorylation rates and the oxidative capacities of rat skeletal mitochondria increased with increasing assay temperature. These observations clearly indicate that muscle temperature could have a strong impact on the functioning of skeletal muscle mitochondria.

Mammalian body is often exposed to both low and high temperatures that lead to the risk of hypothermia or hyperthermia, respectively. Changes in temperature affect muscle endurance performance but the underling mechanisms remain largely unknown. Recent studies suggest that endurance training induces qualitative regulatory changes within mitochondria [28]. Surprisingly, to the best of our knowledge, no data have been published so far that describe the effect of endurance training on the functioning of isolated mitochondria at varied temperature. In the present study, for the first time, we examined the effect of endurance training on respiratory activities in phosphorylating and nonphosphorylating states, the yield of ATP synthesis, mΔΨ, mitochondrial uncoupling (proton leakage), Q level, and ROS production. Moreover, the effect of prolonged endurance training on the mitochondrial oxidative capacity was studied, for the first time, at various temperatures by measuring maximal activities of cytochrome *c* oxidase (COX) and citrate synthase (CS). All the parameters were studied in rat skeletal mitochondria at temperatures of 25, 35, and 42 °C. These temperatures lie in the range of survivable body temperatures for rats (cf. ref. [25]). The highest temperature used in this study (42 °C) is a temperature to which mammalian muscle can increase during exercise [33]. The novelty of the present study lies in demonstrating, for the first time, the effects of endurance training on OXPHOS efficiency, uncoupling and ROS production in skeletal muscle mitochondria at different temperatures. Our results show that the endurance training-induced temperature-dependent changes in muscle mitochondria functioning could be essential to muscle energy metabolism and exercise performance.

## Material and methods

### Chemicals

All chemicals were the highest available grade and were purchased from Sigma-Aldrich unless otherwise mentioned.

### Animals

The study was performed on 24 adult 4-month-old male Wistar rats. Animals were randomly assigned to either a treadmill training group (*n* = 12) or a sedentary control group (*n* = 12). During the experiment, the rats were kept in standard laboratory cages (two per cage), in a room with a 12 h/12 h light/dark cycle, controlled temperature (22 ± 2 °C), and humidity (55 ± 10 %). All rats had unrestricted access to standard rat feed, ensuring a balanced diet and to tap water throughout the study period.

### Animal endurance training

The training, which lasted 8 weeks, was performed at 22 ± 2 °C, five times per week (Monday–Friday) on a standard treadmill for small rodents (Exer 3/6 M Treadmill, Columbus Instruments, Columbus, OH, USA). The running belt was placed horizontally with a 0° inclination. In the first week of the training, the rats were familiarized with running on the treadmill at various velocities (20–30 m min^−1^) during 20–30 min running sessions. At the end of the first week, the duration of a training session was 40 min. In the first 2 weeks of training the basal running velocity set at 30 m min^−1^, but every 10 min, it was increased up to 40 m min^−1^ for approximately 20 s. From the fifth week of training, the duration of training sessions was extended to 60 min. The basal running velocity at this stage of training was set to 30 m min^−1^, and approximately every 10 min the running velocity was increased up to 40 m min^−1^. The duration of the higher speed was gradually increased its duration from 20 s in the sixth week up to approximately 40 s in the final week of training. At the end of the training period, 22–24 h after completing the last training session, the rats were sacrificed by stunning and decapitation and all efforts were made to minimize suffering. No anesthetics were used in order to avoid its impact on the studied mitochondria functioning. The same procedure was applied for sedentary rats (control group). This training schedule was similar to the training protocols described previously [12]. The aim of the training program was to obtain a significant acceleration in the biogenesis of mitochondrial proteins in the locomotor muscles of trained rats. In our study, the 8-week endurance training led to a significant decrease in rat body mass and a significant elevation in muscle mitochondrial yield (Supplementary data, Table S1).

### Skeletal muscle homogenate and mitochondria preparation

Immediately after decapitation of the animals (both control and trained rats), hindlimbs were rapidly removed at the level of the hip joints in order to dissect all major hindlimb locomotor muscles. In order to obtain sufficient mass of muscle tissue to collect adequate amount of mitochondrial fraction, the obtained muscles from shank and thigh were used as a mix muscle sample to obtain muscle homogenates and mitochondria fraction for further measurements as previously described in [25]. To chelate free fatty acids, 0.2 % bovine serum albumin (BSA) was used in an isolation medium. The obtained isolated mitochondria, both from control and trained rats, were quite stable for 6–7 h and their outer mitochondrial membrane exhibited good integrity (97–97 %).

### Protein concentration determination

Muscle homogenate and mitochondrial protein concentration was determined using the Bradford method with BSA as a standard [5].

### Maximal CS and COX activities

The maximal CS and COX activities were assayed in rat skeletal muscle homogenates and isolated mitochondria fractions at various temperatures (25, 35, and 42 °C) as described previously [41]. CS activity was measured with a UV-1650 Shimadzu spectrophotometer at 412 nm with 100 mM 5,5′-dithiobis-(2-nitrobenzoic acid) (TNB) and 40–60 μg of muscle homogenate protein or muscle mitochondrial protein. COX activity was measured polarographically using a Clark-type oxygen electrode (Hansatech), as described previously [25], in 0.7 ml of a respiration medium containing 225 mM mannitol, 75 mM sucrose, 10 mM KCl, 5 mM KH_2_PO_4_, 0.5 mM EDTA, 0.5 mM EGTA, 0.05 % BSA, 10 mM Tris-HCl, and pH 7.2, with 40–60 μg of muscle homogenate protein or muscle mitochondrial protein.

### Measurements of mitochondrial respiration and mΔΨ

Measurements were performed in isolated mitochondria at various temperatures (25, 35, and 42 °C) as described previously [25]. Oxygen uptake was determined polarographically using a Rank Bros. oxygen electrode or a Hansatech oxygen electrode in either 2.8 or 0.7 ml of respiration medium (225 mM mannitol, 75 mM sucrose, 10 mM KCl, 5 mM KH_2_PO_4_, 0.5 mM EDTA, 0.5 mM EGTA, 0.05 % BSA, 10 mM Tris-HCl, pH 7.2), with either 1 or 0.25 mg of mitochondrial protein (0.36 mg ml^−1^). Membrane potential (mΔΨ) was measured simultaneously with oxygen uptake using a tetraphenyl-phosphonium (TPP^+^)-specific electrode. The values of mΔΨ were corrected for TPP^+^ binding using the apparent external and internal partition coefficients of TPP^+^ [39].

Succinate (5 mM) with rotenone (2 μM) or malate (5 mM) plus pyruvate (2 mM) were used as respiratory substrates. Oxidative phosphorylation studies were performed in the absence of Mg^2+^ to avoid the adenine nucleotide interconversion catalyzed by mitochondrial adenylate kinase and to ensure high coupling between electron transport and ATP synthesis. To chelate endogenous free fatty acids in mitochondrial preparations (especially in mitochondria from trained rats), 0.05 % BSA was always present in the respiration medium. Phosphorylating respiration (state 3) was measured after an ADP pre-pulse (50 μM) using 150 μM ADP as a main pulse. The total amount of oxygen consumed during state 3 respiration was used for calculation of the ADP/O ratio. Measurements of mΔΨ allowed for fine control of the duration of state 3 respiration.

The proton-conductance response to a driving force can be expressed as the relationship between the oxygen consumption rate and the mΔΨ (flux/force relationship) when varying the potential by titrating with respiratory-chain inhibitors. Proton leak assessments during nonphosphorylating (resting, state 4) respiration were performed as previously described [25] with 5 mM succinate (plus 2 μM rotenone) as an oxidizable substrate in the absence of exogenous ADP and the presence of 1.8 μM carboxyatractyloside and 0.7 μg/ml (2 μg per mg of protein) oligomycin, which inhibits the activities of the ATP/ADP anti-porter and ATP synthase, respectively. MgCl_2_ (0.5 mM) was added to the respiration medium. To induce uncoupling protein (UCP2/3) activity, linoleic acid (up to 16 μM) was used. To inhibit UCP2/3 activity, 2 mM GTP was added. To decrease the rate of the Q-reducing pathway, succinate dehydrogenase was titrated with malonate (up to 1.7 mM).

### Assay of H_2_O_2_ production by isolated muscle mitochondria

Mitochondrial H_2_O_2_ production was measured using the Amplex Red-horseradish peroxidase method (Invitrogen) [25]. Fluorescence was kinetically followed for 15 min at an excitation wavelength of 545 nm and an emission wavelength of 590 nm using an Infinite M200 PRO Tecan multimode reader that was adjusted internally to different temperatures. Mitochondria (0.1 mg of mitochondrial protein) were incubated in 0.7 ml of the respiration medium (see above) with 5 mM succinate (a FAD-linked oxidizable substrate), 5 mM malate plus 2 mM pyruvate (NAD-linked oxidizable substrates), or 5 mM succinate plus 5 mM malate (FAD- and NAD-linked oxidizable substrates) in the absence of rotenone, oligomycin, carboxyatractyloside, and MgCl_2_. Reactions were monitored under constant stirring. No difference in H_2_O_2_ formation was observed when measurements were performed in the respiration medium containing 300 mM mannitol instead of 225 mM mannitol plus 75 mM sucrose.

### The determination of Q content and reduction level in isolated muscle mitochondria

Total mitochondrial Q9 content and the Q reduction level (the reduced Q vs. the total endogenous pool of Q9) in the inner mitochondrial membrane were determined using an extraction technique followed by HPLC measurements [24]. For calibration of the HPLC peaks, commercial Q9 was used (Sigma-Aldrich).

### Determination of protein levels through immunoblotting

RIPA buffer (150 mM NaCl, 1 % Triton X-100, 0.5 % Na deoxycholate, 0.1 % SDS, 50 mM Tris, pH 8.0) was used to lyse the muscle homogenates. The muscle homogenates and mitochondrial fractions were isolated in the presence of protease inhibitors (Sigma). The proteins were separated on an 8, 10, or 12 % SDS-PAGE gel. The Spectra™ Multicolor Broad Range Protein Ladder (Fermentas) was used as a molecular weight marker. The following primary antibodies were used: anti-citrate synthetase (CS, 52 kDa) (ab-96,600, Abcam), anti-cytochrome *c* subunit II (COXII, 24 kDa) (ab110258, Abcam), anti-β actin (42 kDa) (CP01, Calbiochem), anti-glyceraldehyde-3-phosphate dehydrogenase (GAPDH, 37 kDa) (ab9485, Abcam), anti-mitochondrial transcription factor A (TFAM, 28 kDa) (ab131607), anti-peroxisome proliferator-activated receptor gamma coactivator 1-alpha (PGC1α), anti-mitochondrial Mn superoxide dismutase (SOD2, 25 kDa) (ADI-SOD, Enzo Life Sciences), anti-mitochondrial marker (MTC02, 60 kDa nonglycosylated protein component of mitochondria) (ab3298, Abcam), and the MitoProfile® total OXPHOS human antibody cocktail (MS601, MitoScience) containing antibodies raised against subunit of complex I (20 kDa subunit NDUFB8), complex II (30 kDa subunit), complex III (subunit Core 2, 47 kDa), complex IV (COXII, 24 kDa), and ATP synthase (subunit α, 57 kDa). Appropriate horseradish peroxidase-conjugated secondary antibodies were used. The expression levels of COXII or mitochondrial marker (for the mitochondrial fractions) and of β-actin or GAPDH (for the homogenate fractions) were used as loading normalization controls. Protein bands were visualized using the Amersham ECL system and digitally quantified using the GeneTools 4.03 software package.

### Statistical analysis

The results are presented as the means ± SD obtained from at least six independent muscle homogenate preparations or mitochondrial isolations, in which each determination was performed at least in duplicate. ANOVA followed by a post hoc Tukey’s test for pairwise comparisons was used to identify significant differences. Differences were considered to be statistically significant if *p* < 0.05 (* or ^#^), *p* < 0.01 (** or ^##^), or *p* < 0.001 (*** or ^###^). ^#^, ^##^, and ^###^ indicate comparison between different temperatures within a given group of animals, i.e., within control rats or trained rats. ^*^, ^**^, and ^***^ indicate comparison of data obtained between trained rats vs. control rats. Statistical analysis was performed with Origin v 8.5.1 software (OriginLab Corporation, Northampton, MA).

## Results

### Endurance training magnifies the high external temperature-induced increases in mitochondrial oxidative capacities

**Fig. 1:**
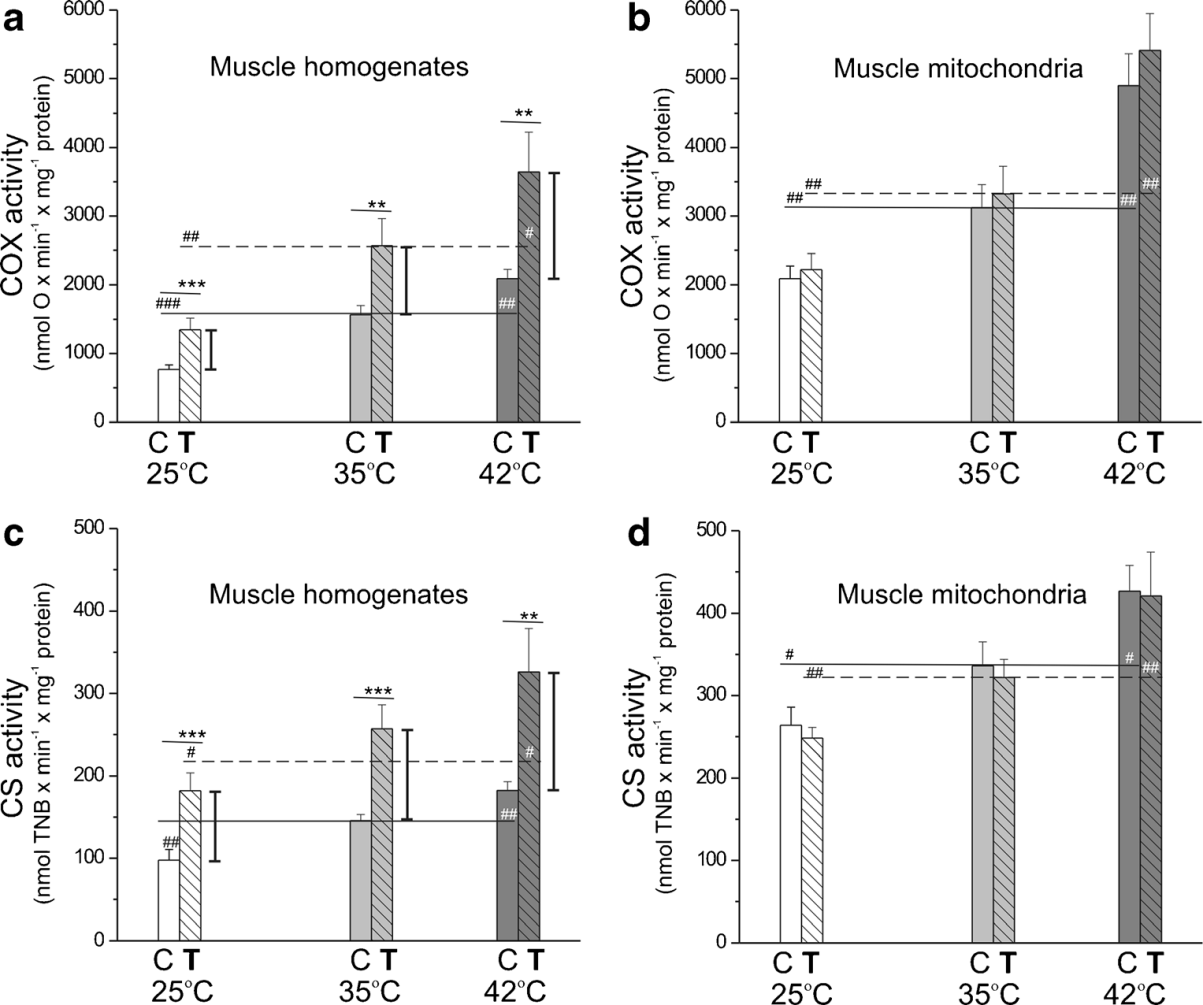
The influence of endurance exercise training on maximal COX (**a**, **b**) and CS (**c**, **d**) activities at 25, 35, and 42 °C. Measurements were performed in skeletal muscle homogenates (**a**, **c**) and isolated mitochondria (**b**, **d**). Data are represented as the mean ± SD (*n* = 18) and are from six independent muscle homogenate preparations or mitochondrial preparations (triplicate assays for each condition). *Number signs*, comparison vs. value obtained at 35 °C for control (*C*) or trained (*T*) group. *Asterisks*, comparison vs. value obtained for control rats. Range of changes between control and trained rats are shown as *vertical lines*

**Fig. 2:**
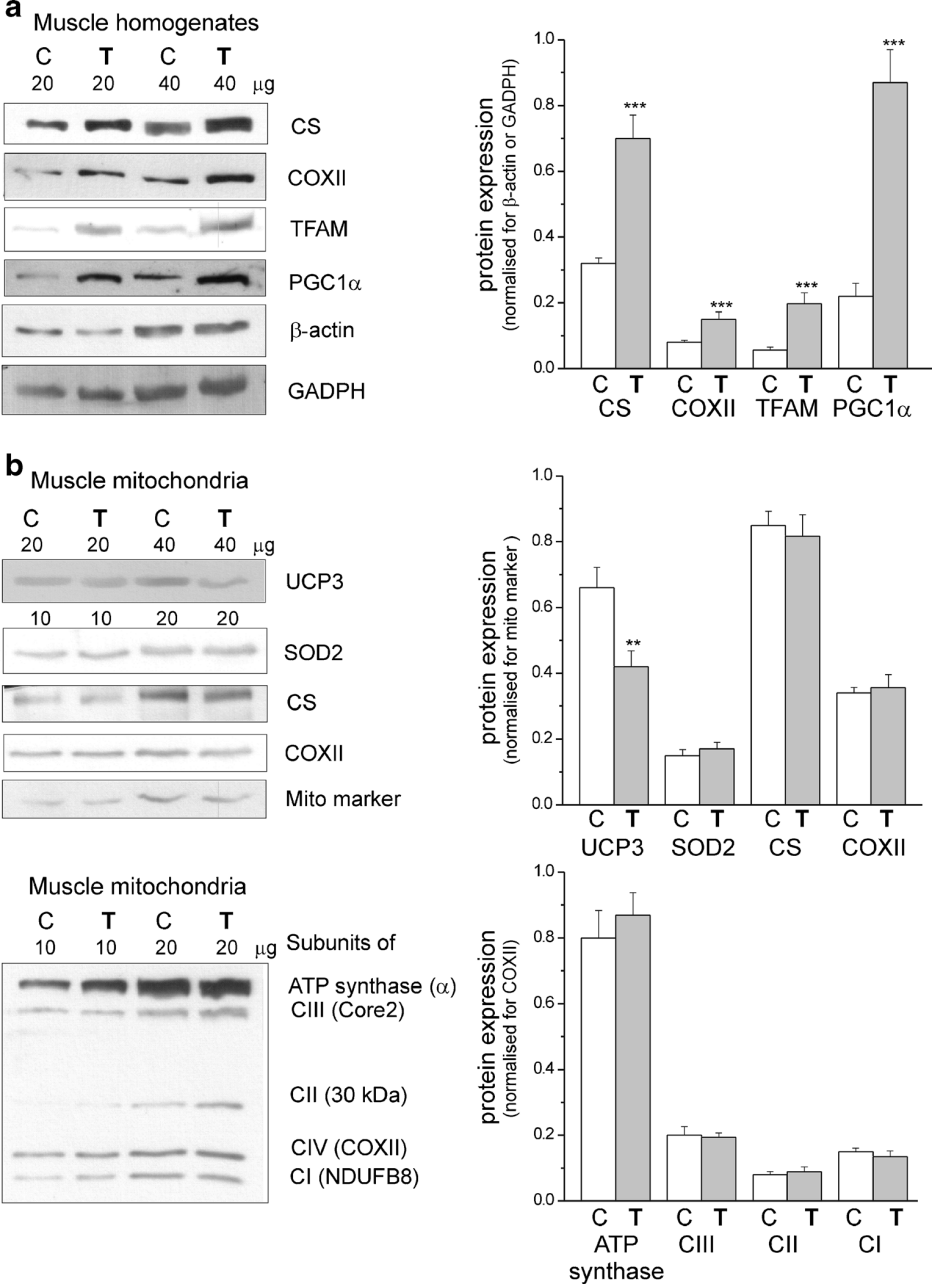
Determination of protein levels in skeletal muscle homogenates (**a**) and mitochondria (**b**) from control (*C*) and trained (*T*) rats. **a** Representative Western blots and analyses of the protein expression of CS, COXII, TFAM, PGC1α, β-actin, and GADPH. **b** Representative Western blots and analyses of the protein expression of UCP3, SOD2, CS, COXII, mitochondrial marker (mito marker), and particular subunits of ATP synthase, complex III (CIII), complex II (CII), and complex I (CI). Expression levels normalized for β actin (**a**), mito marker (**b**, *upper panel*), or COXII (**b**, *lower panel*) protein abundance are shown (**a**, **b**, *right panels*). The data (±SD, *n* = 12) are from six independent homogenate or mitochondrial preparations (duplicate assays for each experiment). *Asterisks*, comparison vs. value obtained for control rats

Mitochondrial oxidative capacities were elucidated by measuring the maximal COX and CS activities in skeletal muscle homogenates (Fig. 1a, c) and isolated mitochondrial fractions (Fig. 1b, d) obtained from control rats and rats trained for 8 weeks. Oxidative capacities were measured at various temperatures. In both muscle homogenates and muscle mitochondria of sedentary and exercising rats, the activities of COX (complex IV of mitochondrial respiratory chain) and CS (a key pace-making enzyme of the tricarboxylic acid (TCA) cycle) significantly increased with increasing assay temperature. In muscle homogenates from exercising rats, but not in isolated mitochondria, an increase in activities of both enzymes was observed compared to those in the control rats, with the most pronounced effects at 42 °C. The increased COX and CS activities (Fig. 1a, c) and their protein levels (Fig. 2a), along with a significantly higher mitochondrial isolation yield (Supplementary data, Table S1), indicate accelerated mitochondrial biogenesis in the skeletal muscle of trained rats. In addition, a significantly higher expression level of other mitochondrial biogenesis markers, TFAM and PGC1α, was detected in the skeletal muscle homogenates of trained rats (Fig. 2a). Moreover, endurance training magnifies the high external temperature-induced increase in the mitochondrial oxidative capacities of the respiratory chain and the TCA cycle.

### In muscle mitochondria, endurance training attenuates the hyperthermia-induced decrease in efficiency of oxidative phosphorylation and augments the hyperthermia-induced increase in phosphorylation rate

**Fig. 3:**
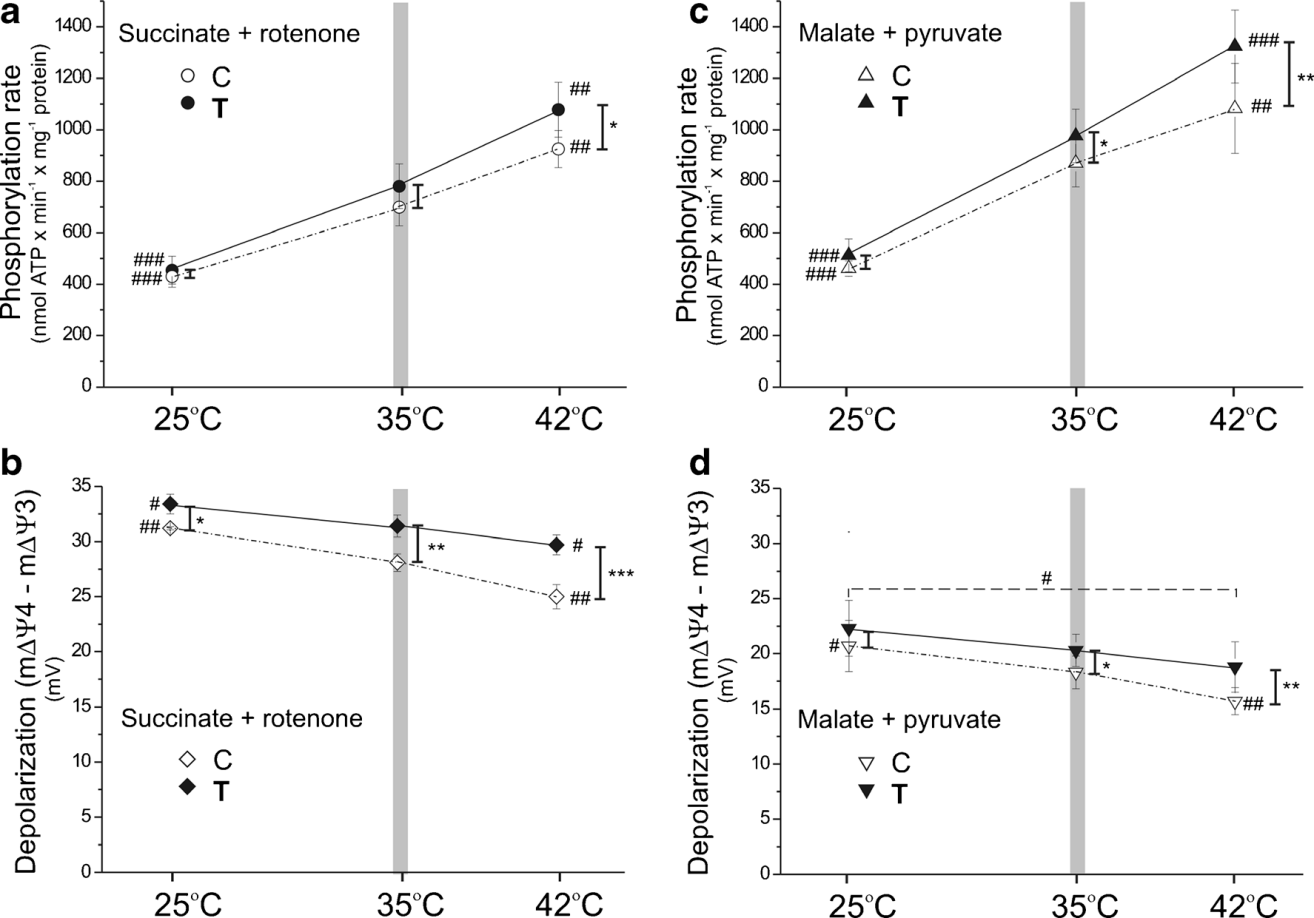
Temperature dependence of the phosphorylation rate and mΔΨ depolarization in skeletal muscle mitochondria isolated from control and trained rats. Experimental conditions are similar to those in Table 1. Measurements were performed with succinate (plus rotenone) (**a**, **b**) or malate plus pyruvate (**c**, **d**) as respiratory substrates. The ADP phosphorylation rate (**a**, **c**) is equal to state 3 respiration × ADP/O. Membrane potential values were measured under state 4 (mΔΨ4) and state 3 (mΔΨ3) conditions. The resulting mΔΨ depolarization (mΔΨ4 minus mΔΨ3) is presented (**b**, **d**). **a**–**d** Every data point represents the mean ± SD (*n* = 27) for at least nine independent mitochondrial preparations, in which every condition was performed in triplicate. *Number signs*, comparison vs. value obtained at 35 °C (a *vertical gray bar*) for control (*C*) or trained (*T*) group. *Asterisks*, comparison vs. corresponding value obtained for control rats. Range of changes between control and trained rats are shown as *vertical lines*

The different parameters of the mitochondrial OXPHOS process were studied for the three tested temperatures in mitochondria isolated from the skeletal muscles of trained and control rats (Table 1; Fig. 3). In both types of mitochondria, with both added respiratory substrates (succinate plus rotenone for complex II and malate plus pyruvate for complex I), the considerable increase in the oxidation rate was observed with increasing temperature in phosphorylating respiration (state 3) and in nonphosphorylating respiration (state 4) (Table 1). Interestingly, in both types of mitochondria, independent of assay temperature, the apparent maximal mitochondrial respiratory chain respiration rate (observed in phosphorylating, succinate-oxidizing mitochondria) (Table 1) composed ∼14–18 % of the apparent capacity of complex IV (COX) (Fig. 1b). For a given assay temperature, no change in phosphorylating respiration or a significant decrease in nonphosphorylating respiration was observed in muscle mitochondria from trained rats compared to those of the controls.

**Table 1:** Respiratory rates, mΔΨ values, and coupling parameters in control and trained rat skeletal muscle mitochondria at 25, 35, and 42 °C

	Control rat mitochondria			Trained rat mitochondria		
Succinate + rotenone	25 °C	35 °C	42 °C	25 °C	35 °C	42 °C
State 3 rate	316 ± 28^###^	568 ± 54	881 ± 78^###^	318 ± 32^###^	587 ± 52	914 ± 89^###^
State 3 mΔΨ	143.1 ± 0.7	142.9 ± 1.5	142.4 ± 1.2	142.4 ± 0.9	142.0 ± 0.8	140.4 ± 1.1
State 4 rate	61.9 ± 5.2^###^	141 ± 7.8	285 ± 28^###^	49.8 ± 5.0^###,^***	125 ± 14*	266 ± 22^###^
State 4 mΔΨ	174.1 ± 1.4^###^	171.2 ± 1.2	167.4 ± 1.5^###^	175.4 ± 1.2^#^	173.8 ± 1.1***	170.1 ± 1.3^###,^**
RCR	5.14 ± 0.36^###^	4.03 ± 0.28	3.11 ± 0.26^###^	6.34 ± 0.59^###,^**	4.70 ± 0.33**	3.43 ± 0.41^###^
ADP/O	1.36 ± 0.05^###^	1.23 ± 0.06	1.05 ± 0.07^###^	1.43 ± 0.07^##,^*	1.33 ± 0.06*	1.18 ± 0.06^###,^*
Malate + pyruvate	25 °C	35 °C	42 °C	25 °C	35 °C	42 °C
State 3 rate	197 ± 12^###^	401 ± 37	605 ± 53^###^	201 ± 18^###^	412 ± 35	613 ± 51^###^
State 3 mΔΨ	142.0 ± 1.8	141.7 ± 1.2	140.9 ± 1.8	141.8 ± 1.6	141.4 ± 1.3	140.8 ± 1.1
State 4 rate	33.7 ± 3.9^###^	88.6 ± 13	168 ± 18^###^	26.8 ± 1.6^###,^***	73.4 ± 8.2**	146.2 ± 13^###,^**
State 4 mΔΨ	162.7 ± 1.4^##^	160.0 ± 1.3	156.6 ± 1.6^###^	164.1 ± 1.2^##,^*	161.7 ± 1.5*	159.6 ± 1.7^#,^**
RCR	5.86 ± 0.43^###^	4.87 ± 0.34	3.63 ± 0.37^###^	6.97 ± 0.63^###,^**	5.64 ± 0.39***	4.12 ± 0.35^###,^**
ADP/O	2.37 ± 0.15^#^	2.17 ± 0.13	1.79 ± 0.20^###^	2.55 ± 0.15^#,^*	2.37 ± 0.16*	2.12 ± 0.13^#,^**

Respiratory rates were measured in the absence (state 4, nonphosphorylating respiration following phosphorylating respiration) or presence (state 3, phosphorylating respiration) of 150 μM ADP with 5 mM succinate (plus 2 μM rotenone) or 5 mM malate plus 2 mM pyruvate. The respiratory rates (in nmol O min^−1^ mg^−1^ protein) and mΔΨ values (in mV) of state 3 and state 4 as well as corresponding respiratory control ratios (RCR) and ADP/O ratios are presented. The RCR is equal to the ratio of state 3 to state 4 respiration. Mean values (±SD) for at least nine different mitochondria preparations (with triplicate measurements, *n* = 27) are shown

*#* comparison vs. value obtained at 35 °C for a given group; * comparison vs. value obtained for control rats

In both types of mitochondria, in contrast to the oxidation rates, the OXPHOS efficiency (ADP/O ratio) and the respiratory control ratio (RCR) decreased significantly with the increasing assay temperature (Table 1), indicating a temperature-induced uncoupling. Independent of temperature, both coupling parameters (ADP/O ratio and RCR) were considerably higher in mitochondria from the muscles of trained rats than from the muscles of control rats.

Figure 3a, c shows that, similar to the pattern observed in oxidation rate, the ATP synthesis rate (ADP phosphorylation rate) was also significantly elevated with the increasing assay temperature in both types of mitochondria and for both added substrates. When comparing both types of mitochondria, at a given assay temperature, the phosphorylation rate was always higher in muscle mitochondria from trained rats. The increase in phosphorylation rate between both types of mitochondria was the most pronounced at 42 °C. In the lowest assayed temperature (25 °C), only a slight increase in the phosphorylation rate was found after training. Taking into account the lack of a significant change in the phosphorylating respiratory rate between muscle mitochondria from control and trained rats (Table 1), the highest increase in ATP synthesis rate observed at 42 °C (Fig. 3a, c) results from the greatest difference in the ADP/O ratio observed between muscle mitochondria from trained and control rats at this temperature (Table 1).

The mΔΨ value reflects substrate oxidation (proton electrochemical gradient producing reactions) and ATP turnover and proton leak (proton electrochemical gradient consuming reactions). The mΔΨ value under nonphosphorylating conditions, as well as the mΔΨ depolarization (change in mΔΨ in response to ADP phosphorylation during the transition from state 4 to state 3 respiration), reflects the level of mitochondrial uncoupling. In muscle mitochondria from control and trained rats, no statistically significant temperature-dependent changes in mΔΨ values were observed for phosphorylating mitochondria for either added respiratory substrate (Table 1). However, in both types of mitochondria, the increase in assay temperature from 25 to 42 °C resulted in a significant decrease in mΔΨ in the nonphosphorylating state (Table 1) and a significant decrease in mΔΨ depolarization (Fig. 3b, d), indicating that the lower ADP/O and RCR ratios observed at the higher temperature (Table 1) are a consequence of mitochondrial uncoupling increasing together with the surrounding temperature. When comparing both types of mitochondria for a given assay temperature, no difference in the mΔΨ of phosphorylating state was accompanied by an increased mΔΨ of nonphosphorylating state (Table 1), resulting in an increased mΔΨ depolarization (Fig. 3b, d) in muscle mitochondria from trained rats compared to those of the controls. The changes observed between mitochondria from trained and control rats were more pronounced with increasing temperature. These observations suggest that endurance training leads to less uncoupling in muscle mitochondria, especially at 42 °C.

Thus, our results indicate that skeletal muscle mitochondria from trained rats compared to mitochondria from sedentary rats show a lower decrease in OXPHOS efficiency and a greater increase in phosphorylation rate at higher temperatures. Thus, endurance training leads to more efficient muscle mitochondrial oxidation and phosphorylation processes, especially at a high external temperature.

### In muscle mitochondria, endurance training did not change the level of the basic protein components of the respiratory chain or of ATP synthase

As described above, the TCA cycle (CS activity) (Fig. 1d) and the maximal cytochrome pathway activity (succinate or malate plus pyruvate oxidation under phosphorylating conditions) (Table 1), as well as maximal COX activity (Fig. 1b), were unaffected in muscle mitochondria after 8 weeks of endurance training. Moreover, the expression levels of the subunits of ATP synthase (α subunits) and the four respiratory chain complexes, namely, complex I (NDUFB8), complex II (subunit 30 kDa), complex III (Core2), and complex IV (COXII), were unchanged in muscle mitochondria as a result of the endurance training (Fig. 2b, lower panels). In addition, no change in the expression level (and activity, Fig. 1d) of CS was observed in muscle mitochondria from trained rats compared to those of control rats (Fig. 2b, upper panels), indicating that endurance training also produced no discernible change at the level of the TCA cycle.

### Endurance training counteracts the high external temperature-induced elevation of ATP turnover-independent proton leakage, including UCP-mediated proton leakage

**Fig. 4:**
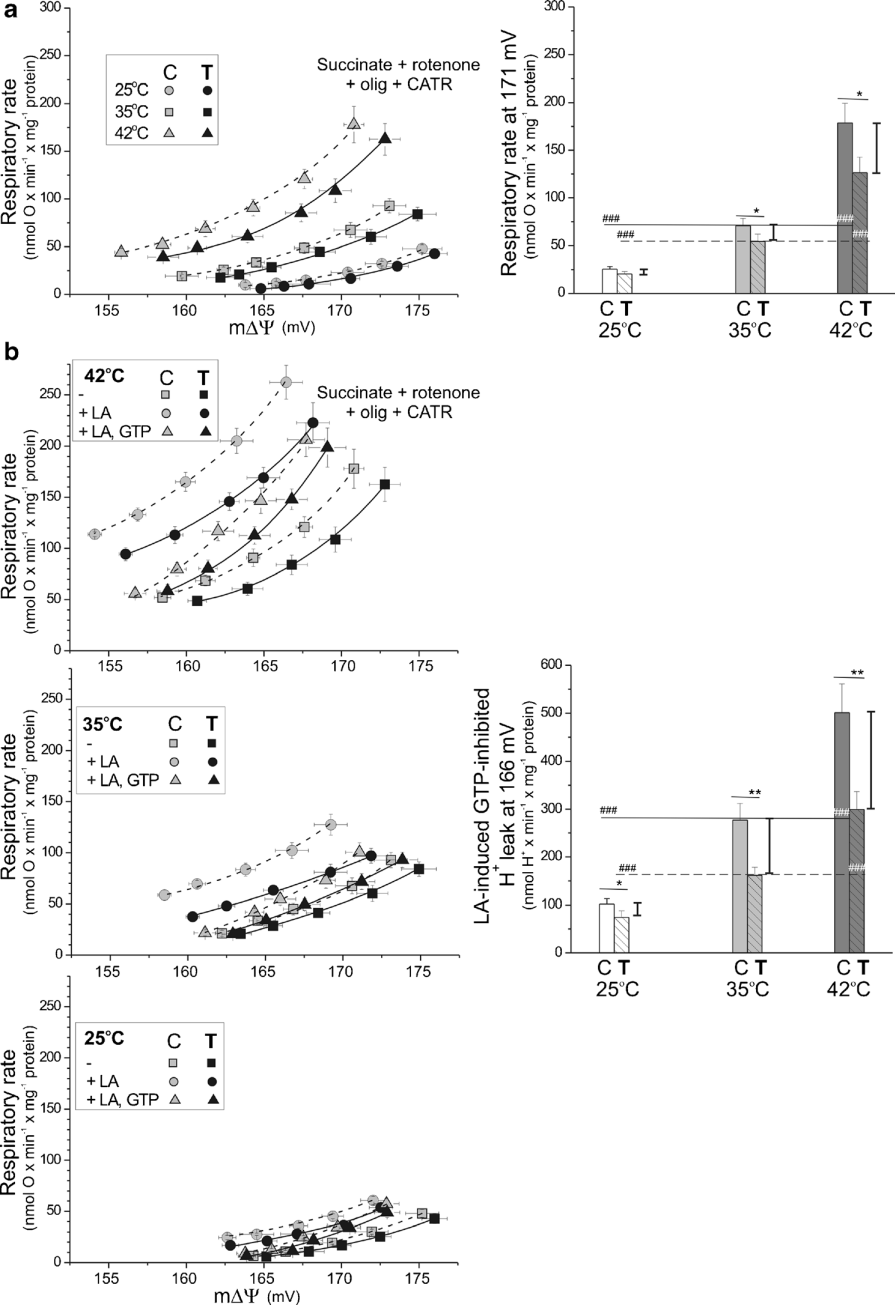
The influence of exercise training on mitochondrial membrane proton conductance at various temperatures in the absence (**a**) or presence (**b**) of UCP modulators. Respiratory rates and mΔΨ were measured simultaneously during progressive inhibition by malonate (up to 1.7 mM) of the respiratory chain oxidizing succinate (5 mM) plus rotenone (2 μM) in the presence of 0.7 μg/ml oligomycin (olig) and 1.8 μM carboxyatractyloside (CATR). Measurements of proton leak kinetics were performed in the absence (**a**) or presence (**b**) of UCP modulators (16 μM linoleic acid, LA) and/or 2 mM GTP) on nonphosphorylating mitochondria incubated at 25, 35, or 42 °C. **a** (*right panel*) Respiratory rates at the highest common mΔΨ value (171 mV) for all tested temperature conditions in mitochondria from control (*C*) and trained (*T*) rats. The data are presented as the means ± SD (*n* = 12) and are from four independent mitochondrial preparations (triplicate assays for each conditions). **b** (*right panel*) Linoleic acid-induced, GTP-inhibited proton leak (UCP activity) at the highest common mΔΨ value (166 mV). Data from representative experiment (mitochondrial preparation) are presented as the means ± SD (*n* = 3) (triplicate assays for each condition). **a**, **b** (*right panels*) *Number signs*, comparison vs. value obtained at 35 °C for control (*C*) or trained (*T*) group. *Asterisks*, comparison vs. corresponding value obtained for control rats. Ranges of changes between control and trained rats are shown as *vertical lines*

A possible decrease in mitochondrial proton leakage (mitochondrial uncoupling) in skeletal muscle mitochondria after endurance training was suggested by the increased OXPHOS efficiency discussed above. The mitochondrial proton electrochemical gradient (with the mΔΨ as the main component) is established by respiratory substrate oxidation and consumed by ATP synthesis and ATP turnover-independent proton leakage. Figure 4 shows the proton conductance kinetics (the relationship between the oxidation rate and the mΔΨ obtained during progressive inhibition of the mitochondrial respiratory chain) in the presence of OXPHOS inhibitors (oligomycin and carboxyatractyloside) as a function of assay temperature in nonphosphorylating muscle mitochondria from control and trained rats. Both inhibitors exclude ATP turnover-dependent proton leakage, and carboxyatractyloside additionally excludes inducible fatty acid-mediated leak through the ATP/ADP anti-porter. In both types of mitochondria, at a higher assay temperature, the relationship between oxygen uptake and mΔΨ was shifted toward a lower mΔΨ along with higher respiratory rate values, indicating increasing proton leak (Fig. 4a). When comparing both types of mitochondria, for a given assay temperature, proton leak was always attenuated in muscle mitochondria from trained rats as indicated by proton leak-driven respiratory rates measured at the highest common mΔΨ value (171 mV) (Fig. 4a, right panel). The difference between mitochondria from trained and control rats was highest at 42 °C. Thus, endurance training leads to a lower proton leak in muscle mitochondria, especially at higher temperatures.

To investigate fatty acid-induced purine nucleotide-inhibited uncoupling protein (UCP)-mediated proton conductance (UCP activity), flux-force relationships were obtained in the presence of a low concentration (16 μM) of linoleic acid and in the absence or presence of 2 mM GTP in nonphosphorylating rat skeletal muscle mitochondria at 25, 35, and 42 °C (Fig. 4b). To exclude inducible fatty acid-mediated leak through the ATP/ADP anti-porter, UCP-mediated uncoupling was studied in the presence of carboxyatractyloside. Because the inhibition of mitochondrial proton conductance by GTP, but not by GDP, can be considered diagnostic of UCP function [40], we used GTP to determine UCP activity in isolated skeletal muscle mitochondria from control and trained rats. In both types of mitochondria, with increasing assay temperature, linoleic acid induced greater mitochondrial uncoupling (a greater increase in oxygen uptake and a greater decrease in mΔΨ), while GTP caused a more pronounced inhibition of this uncoupling (a greater decrease in the oxidation rate and a greater increase in mΔΨ), indicating an elevation of UCP-mediated uncoupling with increasing assay temperature. When comparing both types of mitochondria, for a given assay temperature, UCP-mediated uncoupling was always lower in muscle mitochondria from trained rats than from control rats, as indicated by the linoleic acid-induced, GTP-inhibited proton leak measured at the highest common mΔΨ value (166 mV) (Fig. 4b, right panels). The greatest difference in the linoleic acid-induced, GTP-inhibited proton leak (UCP activity) was observed at 42 °C. Moreover, in response to endurance training, a downregulation of UCP3 protein expression was observed in mitochondria from trained rats (Fig. 2b). Thus, endurance training decreases UCP expression and activity in skeletal muscle mitochondria and attenuates the elevation of UCP-mediated mitochondrial proton leakage resulting from increasing temperature.

Thus, we can conclude that the ATP turnover-independent proton conductance, including UCP-mediated proton leak, decreased in skeletal muscle mitochondria from trained rats. Moreover, endurance training attenuates hyperthermia-elevated mitochondrial proton leakage.

### Endurance training leads to elevated ROS production (and Q reduction level) in nonphosphorylating mitochondria but decreased ROS production (and Q reduction level) in phosphorylating mitochondria

**Fig. 5:**
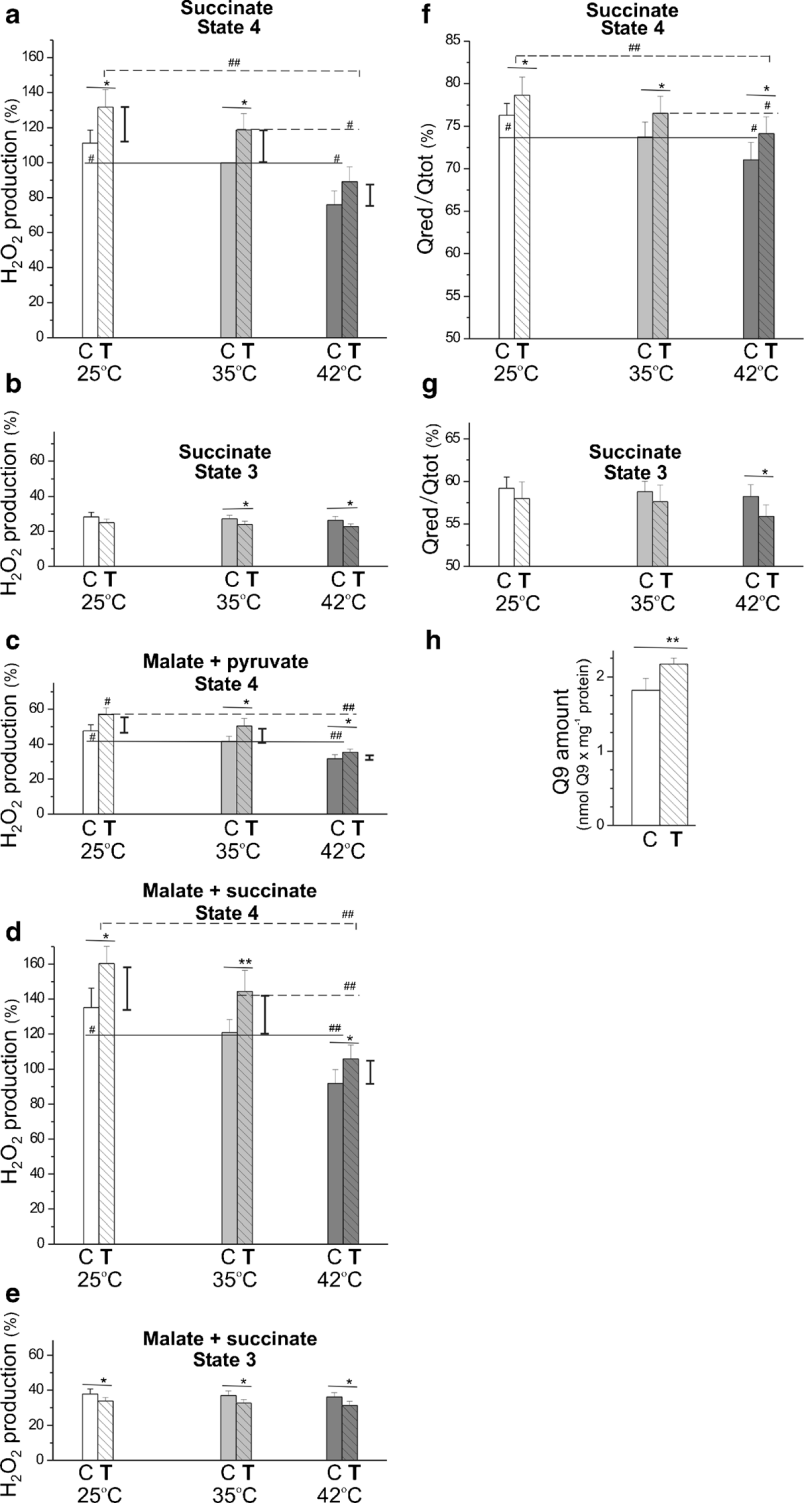
H_2_O_2_ production (**a**–**e**) and Q reduction level (**f**, **g**) in nonphosphorylating and phosphorylating rat skeletal muscle mitochondria isolated from control and trained rats at 25, 35, and 42 °C. **a**–**g** Measurements were performed with 5 mM succinate (in the absence of rotenone), 5 mM malate plus 2 mM pyruvate, and 5 mM malate plus 5 mM succinate in the absence (state 4) or presence (state 3) of 1 mM ADP. **a**–**e** % of H_2_O_2_ formation rate relative to the value obtained with succinate under nonphosphorylating (state 4) conditions at 35 °C for control (*C*) mitochondria (**a**). The data presented as the means ± SD (*n* = 24) are from eight independent mitochondrial preparations (triplicate assays for each experiment). **f**, **g** The data (±SD, *n* = 9) are from three independent mitochondrial preparations (triplicate assays for each experiment). **h** Mitochondrial Q9 content. The data (±SD, *n* = 18) are from six independent mitochondrial preparations (triplicate assays for each experiment). **a**–**g** *Number signs*, comparison vs. value obtained at 35 °C for control (*C*) or trained (*T*) group. *Asterisks*, comparison vs. corresponding value obtained for control rats. For H_2_O_2_ formation measurements in state 4, ranges of changes between control and trained rats are shown as *vertical lines*

A comparison of mitochondrial H_2_O_2_ production under nonphosphorylating (state 4) (Fig. 5a–d) and phosphorylating (state 3) (Fig. 5b, e) conditions indicates that ROS production was higher for nonphosphorylating muscle mitochondria, both from trained and control rats, because the mΔΨ across the inner mitochondrial membrane (Table 1) and the Q reduction level (Fig. 5f, g) were high. Because considerably lower H_2_O_2_ production was observed with complex I substrates (malate plus pyruvate) at a given temperature compared to the complex II substrate (succinate), results for phosphorylating mitochondria were obtained for succinate alone (a FAD-linked substrate) (Fig. 5b) and substrate combination of malate plus succinate (NAD- and FAD-linked substrates) (Fig. 5e). Measurements of H_2_O_2_ formation for nonphosphorylating mitochondria were obtained for succinate alone (Fig. 5a) and substrate combinations of malate plus pyruvate (Fig. 5c) and malate plus succinate (Fig. 5d). When analyzing the effect of temperature on ROS production, a significant decrease in H_2_O_2_ production (and Q reduction level) was observed along with an increase in the assay temperature from 25 to 42 °C in nonphosphorylating mitochondria from trained and control rats, with succinate alone and substrate combinations (Fig. 5a–f). This temperature dependence was not found in both types of mitochondria under phosphorylating conditions (Fig. 5b–g). When comparing both types of mitochondria, mitochondria from trained rats showed a significantly elevated H_2_O_2_ production (and Q reduction level) under nonphosphorylating conditions and a slightly decreased H_2_O_2_ production (and Q reduction level) under phosphorylating conditions compared to those in control rats, independent of assay temperature (Fig. 5). Interestingly, for both complex I and complex II substrates alone and their combinations, the smallest difference (the smallest increase) in H_2_O_2_ production under nonphosphorylating conditions between both types of mitochondria was observed at 42 °C.

Moreover, the Q9 content (the predominant form of Q in rat muscles) was found to be significantly elevated in the muscle mitochondria of trained rats (Fig. 5h). As explained in the “Discussion” section, this change in mitochondrial Q9 content may account for the decreased Q reduction level under phosphorylating conditions (Fig. 5g) and may contribute to the attenuation of the elevated Q reduction level under nonphosphorylating conditions (Fig. 5f).

Thus, in rat skeletal muscle mitochondria, endurance training diminished ROS formation to a similar degree at all assayed temperatures in the phosphorylating state and elevated ROS formation, with smallest increase at 42 °C, in the nonphosphorylating state. Moreover, in the muscle mitochondria of trained rats, a lack of upregulation of mitochondrial antioxidant proteins was observed (Fig. 2b). Namely, endurance training led to a decrease in UCP3 expression level and no significant change in protein expression level of mitochondrial superoxide dismutase (SOD2).

## Discussion

In the present study, an 8-week endurance training resulted in an enhancement of mitochondrial biogenesis, as shown by an increase (∼30 %) in mitochondrial isolation yield from the trained muscles (Supplementary data, Table S1) and a significant increase in the maximal COX (∼65–75 %) and CS (∼60–70 %) activities (Fig. 1a, c), as well as the levels of these proteins and other markers of mitochondrial biogenesis in the muscles of trained rats (Fig. 2a). These observations are in agreement with the results of Holloszy [20] and others [4, 13, 21, 30], showing that prolonged endurance training increases muscle mitochondrial biogenesis. The results presented in this study indicate that endurance training caused not only quantitative mitochondrial changes in the muscle but also a number of qualitative changes in mitochondrial bioenergetic functioning, which differs depending on temperatures.

**Fig. 6:**
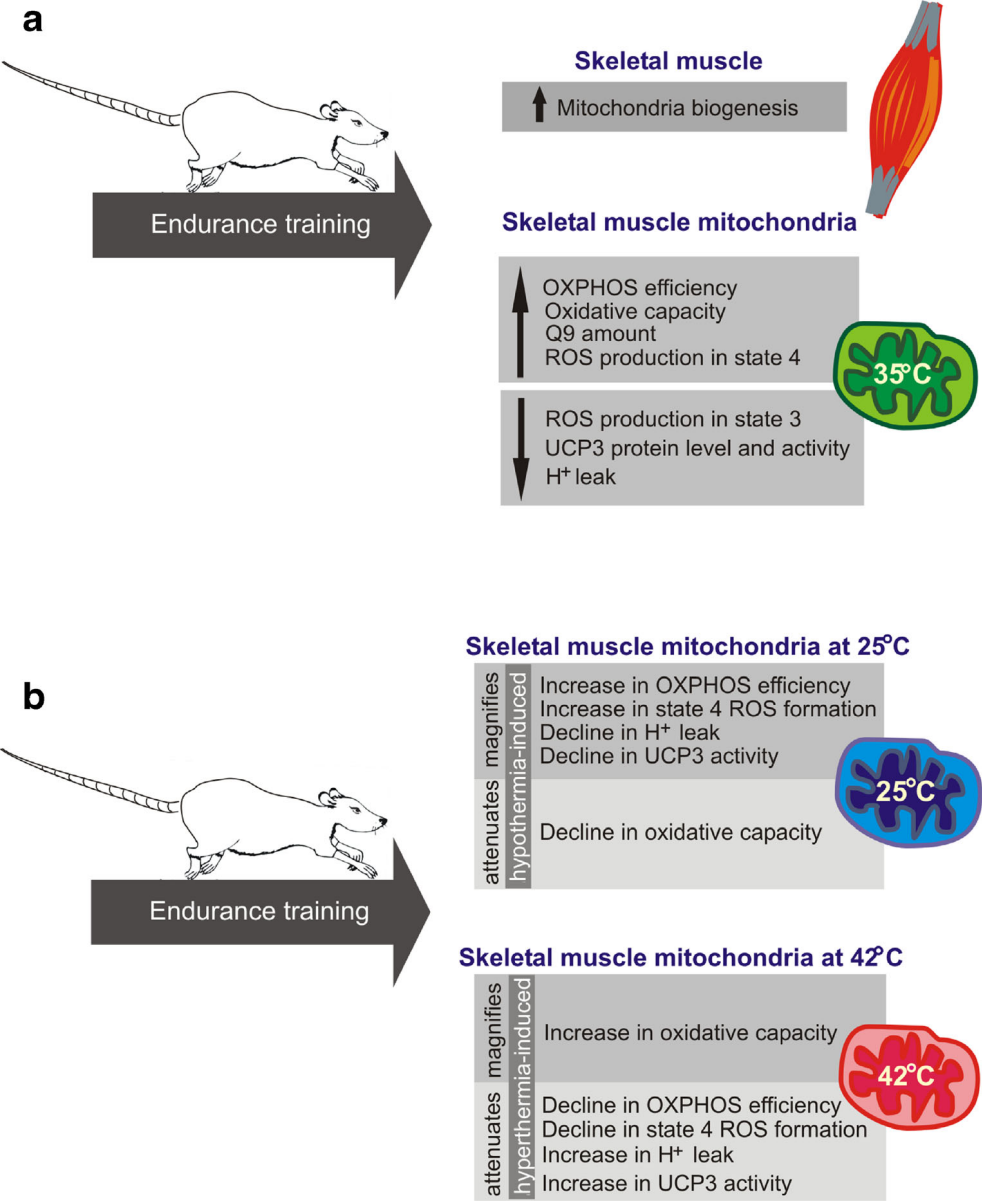
The influence of endurance training on rat skeletal muscle mitochondria functioning (at control temperature 35 °C) and biogenesis (**a**). The effect of endurance training on mitochondrial function at 25 and 42 °C (**b**)

### The impact of endurance training on phosphorylation rate at various temperatures

We have found that the applied endurance training significantly increased the phosphorylation rate, expressed in nmol ATP min^−1^ mg mitochondrial protein^−1^. The new and important finding of our study is that the magnitude of the training-induced increase of mitochondrial phosphorylation capacity varied with assay temperature. Namely, at the highest applied temperature (42 °C), the phosphorylation rate increased by 17–22 % after training, whereas at the lowest temperature (25 °C), only a slight increase (6–10 %) in the phosphorylation rate was found after training (Fig. 3a, c). Taking into account an approximately 60 % augmentation of mitochondrial biogenesis (i.e., the increase in the COX and CS muscle activities; Fig. 1a, c), the overall training-induced increase in mitochondria phosphorylation capacity in muscle could be much higher but still larger in hyperthermia than hypothermia. Thus, our results illustrate that the beneficial effect of endurance training on mitochondrial phosphorylation rate is much more pronounced in hyperthermia than in hypothermia.

### The influence of endurance training on mitochondrial OXPHOS efficiency at various temperatures

Interestingly, at all studied temperatures, the increase in mitochondrial phosphorylation rate was mainly caused by the training-induced enhancement of the mitochondrial OXPHOS efficiency (ADP/O ratio). The magnitude of the ADP/O ratio has a significant impact on muscle mechanical efficiency and physical performance during sustained exercise (for review see [19]) because it determines the amount of ATP generated per unit O_2_ uptake at the mitochondrial level. In isolated mitochondria, the ADP/O ratio is expressed as the number of ADP phosphorylated to ATP when two electrons reduce one O. It has been demonstrated that an increase in assay temperature from normothermia to hyperthermia significantly decreases ADP/O ratio in isolated rat skeletal muscle mitochondria [6, 25, 38]. A similar pattern was observed in the present study both in mitochondria from untrained and 8-week endurance-trained rat locomotor muscles (Table 1). However, reports on the effect of endurance training on the ADP/O ratio in isolated skeletal muscle mitochondria remain unclear. Some studies suggest that different types of endurance training lasting 4–6 weeks essentially have no effect on the ADP/O ratio in human quadriceps muscle [2, 36]. However, it should be noted that the duration of training in these studies was much shorter than in our study, especially when related to the lifespan of the species studied. On the other hand, a decrease in the ADP/O ratio has been observed after 24 h of ultra-endurance exercise in mitochondria from human skeletal muscle [15]. However, other data are consistent with the endurance training-induced improvement of mitochondrial OXPHOS efficiency (elevated ADP/O ratio and RCR) observed in the present study (Table 1). It has been reported that in human skeletal muscle mitochondria, endurance training decreases the magnitude of uncoupled respiration, with nonphosphorylating (state 4) respiration decreasing by ∼20 % [16]. Similarly, an increased energy coupling (measured as elevated ATP production per unit mitochondrial content) has been observed in human skeletal muscles after endurance training and has been postulated to be a factor responsible for enhancing exercise performance of elderly subjects [9]. Moreover, it has been reported previously that the ADP/O ratio in mitochondria from oxidative type I muscle fibers is ∼18 % higher than in those from glycolytic type II muscle fibers [38]. Therefore, the endurance training-induced transformation of fast glycolytic type II muscle fibers into slow oxidative type I muscle fibers [3] could contribute to the observed results in our study showing an increase in the ADP/O ratio after training. The training-induced increase in mitochondrial OXPHOS efficiency (ADP/O ratio) illustrates how mitochondrial ATP production can be upregulated for a given volume of mitochondria after training. However, it seems that the effect of endurance training on mitochondrial OXPHOS efficiency depends on the type and intensity of training applied and its duration with reference to the lifespan of the studied species.

In the present study, the training-induced increase in ADP/O ratio was significant at all assayed temperatures. However, the greatest improvement (∼20 %) was observed in the mitochondria from the trained muscles when assayed at 42 °C with malate plus pyruvate as respiratory substrates (Table 1). Thus, our study shows that the training resulted in an attenuation of the hyperthermia-induced decrease in the mitochondrial OXPHOS efficiency. Therefore, the endurance training could attenuate the magnitude of hyperthermia-induced fatigue by partially restoring the hyperthermia-induced decrease in the ADP/O ratio. This adaptive response could also contribute to the attenuation of the magnitude of the slow component of V′O_2_ on-kinetics [26, 42] and to the decrease of the oxygen cost during high intensity exercise [29] as observed after a few weeks of endurance training.

### The effect of endurance training on mitochondrial uncoupling at various temperatures

In the present study at all studied temperatures, the training-induced increase in OXPHOS efficiency was accompanied by a decline in mitochondrial ATP-independent uncoupling (20, 23, and 29 % at 25, 35, and 42 °C, respectively) and in UCP-mediated uncoupling (27, 42, and 60 % at 25, 35, and 42 °C, respectively) in skeletal muscle mitochondria from trained rats (Fig. 4). In addition to decreased UCP activity, a significant decrease in the UCP-3 protein level (∼44 %) was observed in mitochondria from trained rats (Fig. 2b). A training-induced decrease in UCP3 protein level has been previously found in skeletal muscle mitochondria of humans [16, 32, 35]. However, in our study, when considered together with the ∼60 % increase in mitochondrial biogenesis in trained rat muscles (based on the increase in the CS/COX activities in muscle homogenates; Fig. 1a, c), the training-induced attenuation in overall mitochondrial uncoupling in muscle (∼30 %) takes place only at 42 °C (i.e., at a high temperature that can be reached in muscle during exercise [33]). At 25 and 35 °C, the overall mitochondrial uncoupling in muscle (including UCP-mediated uncoupling) is maintained or slightly increased at the lower temperature as a result of the exercise-induced elevation in mitochondria biogenesis. These measurements are, to the best of our knowledge, the first to estimate the effects of endurance training on muscle mitochondrial uncoupling (ATP-independent and UCP-mediated uncoupling) including changes at various temperatures. Previous studies have shown increases in muscle UCP-3 protein level as a component of the exercise-induced increases in mitochondrial biogenesis [27]. Moreover, our results showed that endurance training attenuated the hyperthermia-elevated mitochondrial proton leakage (uncoupling), which could be beneficial for muscle performance by decreasing oxygen cost during exercise (the V′O_2_/power output ratio). For example, an inverse relationship between UCP3 expression and mechanical efficiency suggests that exercise training produces an adaptive physiological response in skeletal muscle improving mechanical efficiency [35]. In our study, the training-induced decrease in mitochondrial uncoupling (including UCP-mediated proton leak) indicates a higher need for an increase in ATP synthesis yield than for preservation of ROS production. Attenuation of mitochondrial proton leak contributes to the enhancement of ROS production (but only in nonphosphorylating mitochondria) and OXPHOS efficiency.

### The effect of endurance training on mitochondrial coenzyme Q content and reduction level

Although the expression levels of ATP synthase and the four respiratory chain complexes were not affected in rat muscle mitochondria by endurance training (Fig. 2c), the Q9 content was found to be significantly increased (Fig. 5h). This change may account for the decreased Q reduction level observed under phosphorylating (state 3) conditions (Fig. 5g) when considered with similar Q-reducing dehydrogenase activities and Q-oxidizing cytochrome pathway-mediated activity (Table 1). The increase in mitochondrial Q content may also contribute to the attenuation of the elevated Q reduction level under nonphosphorylating (state 4) conditions (Fig. 5f) when considered with the decreased Q-oxidizing activity resulting from lower mitochondrial uncoupling (Fig. 4), which was observed in muscle mitochondria from trained rats. Our findings, for the first time, correlate changes in Q content and Q reduction level of skeletal muscle mitochondria with endurance training. Mitochondrial Q (coenzyme Q), an essential electron carrier of the respiratory chain, is not only critically involved in mitochondrial ROS production but is also an important antioxidant in the mitochondrial inner membrane [23]. Thus, a higher endurance training-induced mitochondrial content of Q (Fig. 5h) may contribute to a decline of Q reduction level (thus a decline in ROS production) in phosphorylating mitochondria and to the attenuation of the increase in Q reduction level (thus the attenuation of ROS production) in nonphosphorylating mitochondria. Finally, this change in Q content may also indicate an increased need for the lipid-soluble antioxidant to act as a protective response to excessive oxidative stress, and/or it may contribute to the higher OXPHOS system efficiency observed in mitochondria from trained rats.

### The effect of endurance training on mitochondrial ROS production at various temperatures

It is well documented that skeletal muscle contractions enhance ROS production [1, 10, 11] and that an increase in ROS can be harmful to muscle cells functioning [1], although in view of the recent studies, this issue seems to be more complex [8]. There is also a growing body of evidence that ROS play an important regulatory role in maintaining muscle homeostasis and in muscle adaptation to exercise, including an intensification of mitochondrial biogenesis (for review see [22]). Although it is thought that in contracting muscle fibers, mitochondria are not the primary source of ROS production, they do seem to play an important role in muscle functioning [25].

The effect of endurance training on mitochondrial ROS production in skeletal muscles is not clear. Studies have reported either an increase [17] or a decrease [18, 37] in mitochondrial H_2_O_2_ formation after training. In the present study, for the first time, we examined the effect of endurance training on H_2_O_2_ production in phosphorylating (state 3) and in nonphosphorylating (state 4) isolated skeletal muscle mitochondria studied in experimentally induced hypothermia (25 °C), normothermia (35 °C), and hyperthermia (42 °C). In general, H_2_O_2_ production, expressed per milligram of mitochondrial protein, was about threefold to fivefold higher in state 4 respiration with succinate or malate plus succinate than in state 3 respiration at a given temperature in mitochondria from both trained and untrained rats (Fig. 5a–e). These phenomena could be explained by a higher proton motive force and a higher Q reduction level present in state 4 respiration than in state 3 respiration, resulting in greater rate of mitochondrial ROS formation. In the present study, a decline of mitochondrial H_2_O_2_ production with increasing assay temperature was observed in both types of rat skeletal muscle mitochondria, especially in nonphosphorylating mitochondria (Fig. 5a–d). Changes in H_2_O_2_ production were accompanied by changes in Q reduction level as estimated for succinate oxidation. For all temperatures, the applied endurance training significantly decreased H_2_O_2_ production (∼13 %) (and Q reduction level) in phosphorylating mitochondria and augmented H_2_O_2_ production (∼18 %) (and Q reduction level) in nonphosphorylating mitochondria. However, in nonphosphorylating mitochondria, the lowest training-induced absolute increase in H_2_O_2_ production was observed at 42 °C. As a result, during succinate oxidation, the state 4/state 3 ratio of H_2_O_2_ production was always higher in mitochondria from trained rats (5.3, 5.0, and 3.9 at 25, 35, and 42 °C, respectively) compared to those from untrained rats (3.9, 3.7, and 2.9 at 25, 35, and 42 °C, respectively) and declined with increasing temperatures. Our results clearly indicate that the lowest ROS production is observed at 42 °C, the temperature of intensely working muscle. Moreover, the training-induced enhancement of ROS is lowest under hyperthermia.

It appears that endurance training could lead to no change or no significant increase in overall mitochondrial ROS production as in the mitochondria of more often working trained muscles (thus more often phosphorylating); a decrease in ROS production in phosphorylating state could be predominant than an elevation of ROS production in nonphosphorylating state. This conclusion is supported by a lack of upregulation of mitochondrial antioxidant proteins in muscle mitochondria of trained rats (Fig. 2b). Specifically, endurance training leads to a decrease in UCP3 expression and no significant change in the protein expression of mitochondrial superoxide dismutase (SOD2). Thus, the likely oxidative stress induced by the applied endurance training was below the threshold needed to induce an enhancement, other than a change in Q level, of the mitochondrial antioxidant system.

Assuming that ROS affect muscle functioning and operate as an important signaling factor in muscle adaptation to exercise [22], the training-induced decrease in ROS production in phosphorylating mitochondria, which predominate in working muscles [7], might contribute to the improvement of muscle functioning during exercise, whereas the training-induced increase in ROS production in nonphosphorylating mitochondria, which predominate in resting muscles [7], might operate as an important regulatory signal for muscle remodeling, including the enhancement of mitochondria biogenesis and the improvement of mitochondria functioning.

It should be kept in mind that rat hindlimb muscles are composed of functionally and metabolically different muscle groups containing various types of muscle fibers [12, 14, 34]. In the present study, we examined the effects of endurance training on functional characteristics of mitochondria isolated from the main rat hind limb muscles representing various muscle groups. Therefore, the reported changes represent adaptive responses that probably take place in muscle fibers, which are the most activated and the most sensitive to endurance training [12]. Most likely, in some of the hind limb muscle groups, the impact of applied endurance training on the studied functional capacity of mitochondria was even greater than that reported in the present paper. Nevertheless, our study clearly shows that an 8-week endurance training is potent to enhance both mitochondrial biogenesis and efficiency in the trained locomotor muscles. In the future, it would be interesting to characterize the magnitude of the impact of endurance training on mitochondrial biogenesis and function in particular locomotor muscle groups of rats and humans.

As summarized in Fig. 6, we found that in rat skeletal muscles, an 8-week endurance training program produced a considerable elevation of mitochondrial biogenesis, which was accompanied by several qualitative temperature-dependent changes at the mitochondrial level. We showed that endurance training was potent to enhance mitochondrial efficiency, especially in hyperthermia, and it had a strong impact on mitochondrial ROS production in relation to mitochondrial energetic status. Endurance training significantly augmented H_2_O_2_ production in nonphosphorylating mitochondria and decreased H_2_O_2_ production in phosphorylating mitochondria. These changes may be critical for maintaining muscle cell energy homeostasis and establishing the training-induced enhancement of exercise performance, especially at high temperatures. Moreover, in addition to providing some new basic knowledge on muscle and exercise physiology, the presented results may put new light on several aspects of functioning of skeletal muscle mitochondria at various temperatures, which may be also important for clinical practice.

## Electronic supplementary material

Supplementary Table 1 (DOC 14.0 kb)
