# Supplementary material for: Endurance training increases the efficiency of rat skeletal muscle mitochondria
Source: Pflugers Arch. 2016 Aug 27;468(10):1709–24. doi: 10.1007/s00424-016-1867-9 (PMC5026720; doi:10.1007/s00424-016-1867-9)
Supplement: Supplementary file 1 — (DOC 14.0 kb) [file 424_2016_1867_MOESM1_ESM.doc]

**Supplementary Table S1**

Eight-week endurance training leads to a significant decrease in rat body mass and a significant elevation of muscle mitochondrial yield.

|  | Control rats | Trained rats |
| --- | --- | --- |
| Animal body mass (g) | 501.8 ± 20.7 | 406.7 ± 24.7*** |
| Mitochondria yield per g of muscle (mg of protein/g of muscle) | 0.76 ± 0.08 | 0.99 ± 0.12*** |

Data are represented as the mean ± SD (*n* = 12 per group). ****p* < 0.001 vs. control rats.
